# Supplementary material for: Predictive utility of the C-reactive protein to albumin ratio in early allograft dysfunction in living donor liver transplantation: A retrospective observational cohort study
Source: PLoS One. 2019 Dec 10;14(12):e0226369. doi: 10.1371/journal.pone.0226369 (PMC6903745; doi:10.1371/journal.pone.0226369)
Supplement: S1 Table — (DOCX) [file pone.0226369.s001.docx]

**Supporting information**

**S1 table.** Postoperative outcomes of patients with the CRP/ALB ratio ≤ and >20%

| **Group** | **CRP/ALB ratio ≤20%** | **CRP/ALB ratio >20%** | ***p*** |
| --- | --- | --- | --- |
| **n** | **340** | **256** |  |
| Hospital stay (day) | 25 (21 – 34) | 28 (21 – 43) | 0.006 |
| During follow-up period |  |  |  |
| eGFR based kidney function grade |  |  | <0.001 |
| Stage 1 | 263 (77.4%) | 144 (56.3%) |  |
| Stage 2 | 50 (14.7%) | 44 (17.2%) |  |
| Stage 3a | 11 (3.2%) | 23 (9.0%) |  |
| Stage 3b | 11 (3.2%) | 31 (12.1%) |  |
| Stage 4 | 3 (0.9%) | 10 (3.9%) |  |
| Stage 5 | 2 (0.6%) | 4 (1.6%) |  |
| Ascites | 107 (31.5%) | 88 (34.4%) | 0.454 |
| Infection | 25 (7.4%) | 32 (12.5%) | 0.034 |
| Graft rejection | 72 (21.2%) | 44 (17.2%) | 0.223 |
| *de novo* Cancer occurrence | 26 (7.6%) | 18 (7.0%) | 0.776 |
| Re-transplantation | 5 (1.5%) | 19 (7.4%) | <0.001 |
| Overall patient mortality | 35 (10.3%) | 55 (21.5%) | <0.001 |

**Abbreviations:** EAD, early allograft dysfunction; eGFR, estimated glomerular filtration rate

**NOTE:** Values are expressed as median (interquartile) and number (proportion).
